# Explaining Local-Scale Species Distributions: Relative Contributions of Spatial Autocorrelation and Landscape Heterogeneity for an Avian Assemblage

**DOI:** 10.1371/journal.pone.0055097

**Published:** 2013-02-05

**Authors:** Brady J. Mattsson, Elise F. Zipkin, Beth Gardner, Peter J. Blank, John R. Sauer, J. Andrew Royle

**Affiliations:** 1 Western Ecological Research Center, United States Geological Survey, Sacramento, California, United States of America; 2 Patuxent Wildlife Research Center, United States Geological Survey, Laurel, Maryland, United States of America; 3 Department of Forestry and Environmental Resources, North Carolina State University, Raleigh, North Carolina, United States of America; Utah State University, United States of America

## Abstract

Understanding interactions between mobile species distributions and landcover characteristics remains an outstanding challenge in ecology. Multiple factors could explain species distributions including endogenous evolutionary traits leading to conspecific clustering and endogenous habitat features that support life history requirements. Birds are a useful taxon for examining hypotheses about the relative importance of these factors among species in a community. We developed a hierarchical Bayes approach to model the relationships between bird species occupancy and local landcover variables accounting for spatial autocorrelation, species similarities, and partial observability. We fit alternative occupancy models to detections of 90 bird species observed during repeat visits to 316 point-counts forming a 400-m grid throughout the Patuxent Wildlife Research Refuge in Maryland, USA. Models with landcover variables performed significantly better than our autologistic and null models, supporting the hypothesis that local landcover heterogeneity is important as an exogenous driver for species distributions. Conspecific clustering alone was a comparatively poor descriptor of local community composition, but there was evidence for spatial autocorrelation in all species. Considerable uncertainty remains whether landcover combined with spatial autocorrelation is most parsimonious for describing bird species distributions at a local scale. Spatial structuring may be weaker at intermediate scales within which dispersal is less frequent, information flows are localized, and landcover types become spatially diversified and therefore exhibit little aggregation. Examining such hypotheses across species assemblages contributes to our understanding of community-level associations with conspecifics and landscape composition.

## Introduction

Understanding linkages between spatial patterns of biological communities and environmental characteristics is a central question in ecology and natural resource conservation [1]–[3]. Lichstein et al. [4] distinguished ‘endogenous’ and ‘exogenous’ factors as potential drivers of species distributions, and this typology is useful to articulate hypotheses for interactions between species settlement patterns and their environment. Endogenous factors themselves could be classified as behavioral decisions or evolutionary constraints [4]–[6]. An important evolutionary constraint for species distributions is dispersal limitation, which could induce spatial aggregation of species [7]–[9]. Likewise, a critical behavioral decision for mobile conspecific individuals (henceforth, conspecifics) is whether to aggregate as a strategy to diversify genetic transfers, enhance foraging efficiency, or to gain safety from predators [10], [11]. In a heterogeneous environment, these endogenous factors could interact with exogenous factors, which would include particular habitat conditions such as landform, microclimate, and vegetation structure and composition that favor fitness [4]–[6]. Distinguishing and accounting for these sources of variability in predicting species distributions remains a formidable challenge.

There are at least four hypotheses that can explain the distribution patterns of species at a given resolution. First, a null hypothesis is that species are distributed randomly and are therefore equally likely to occur among patches, i.e., spatial units at the scale of an individual home range. With such a random distribution, we would predict that a species occurrence pattern corresponds with neither the condition of patches (e.g., local land cover) nor conspecific occupancy of adjacent patches. Although this null hypothesis contradicts much of modern ecological theory [12], [13], this pattern may be more parsimonious if both endogenous and exogenous factors under consideration have only a weak influence. This null hypothesis therefore may serve as a useful baseline for comparison with hypotheses that assume nonrandom species distributions. A second hypothesis is that species are not randomly distributed and that endogenous factors dominate, where the species aggregates such that it is more likely to occur when a patch is surrounded by patches occupied by conspecifics, giving rise to a patchy or regular distribution [14]. Under this aggregation hypothesis, we would predict a positive relationship between patch occupancy and the proportion of adjacent patches that are occupied but a weak or absent relationship with local landcover. Third, heterogeneity in local landcover alone drives the spatial distribution of the species. Based on this land-cover hypothesis, we expect that exogenous local landcover characteristics override conspecific aggregation in driving settlement patterns. Certainly, landcover and environmental variables at other scales can influence species distributions, but here we focus on this local landcover hypothesis. Finally, a global hypothesis is that conspecifics not only aggregate, but they do so also in accordance with local landcover characteristics and their distribution therefore reflects both exogenous and endogenous factors to varying degrees.

As mobile and readily observed animals, breeding birds provide an excellent opportunity for examining hypotheses about species distributions [15], [16]. Furthermore, birds can be sampled efficiently as an entire community, providing a wealth of information with which to confront these hypotheses [17]. Here, we consider a case study on the breeding bird community of the Patuxent Research Refuge located in the Mid-Atlantic region of the United States. This region hosts a diversity of avian guilds that occupy upland forests, lowland forests, wetlands, upland meadows, shrub-scrub, and developed areas [16]. Land managers in the Mid Atlantic are interested in predicting bird species distributions with respect to local conspecific aggregations and land-cover characteristics for developing conservation plans [18], and understanding the relative roles of exogenous landscape context and endogenous species aggregation [19]. This investigation therefore has potential for not only increasing our basic understanding of drivers for species distribution patterns but also for broad applicability across conservation areas in the region.

Our objectives are to 1) ascertain the relative importance of conspecific-neighborhood effects reflecting endogenous drivers vs. landcover variables reflecting exogenous drivers of bird species distributions, and 2) improve understanding about how accounting for conspecific-neighborhood effects impacts inferences about landcover drivers of bird species and community distribution within a forested landscape. We consider an avian point-count dataset from the Patuxent Research Refuge as a case study to analyze patterns in species occupancy while accounting for heterogeneity in detectability among species using a hierarchical Bayes approach. In doing so, we account for possible species-specific associations with particular landcover metrics and spatial autocorrelation. We also account for similarities among species in the community by sampling species-specific parameters from a common hyper-distribution while accounting for detectability [20]. Ignoring heterogeneity in detectability among species and habitats can greatly confound inferences about drivers of species distributions [21], [22]. We then compare our findings with existing literature on species distributions to shed light about the independent and combined strengths of landcover and spatial autocorrelation as sources of heterogeneity for explaining individual species distributions for the entire avian community.

## Methods

### 1. Study Area

**Figure 1:**
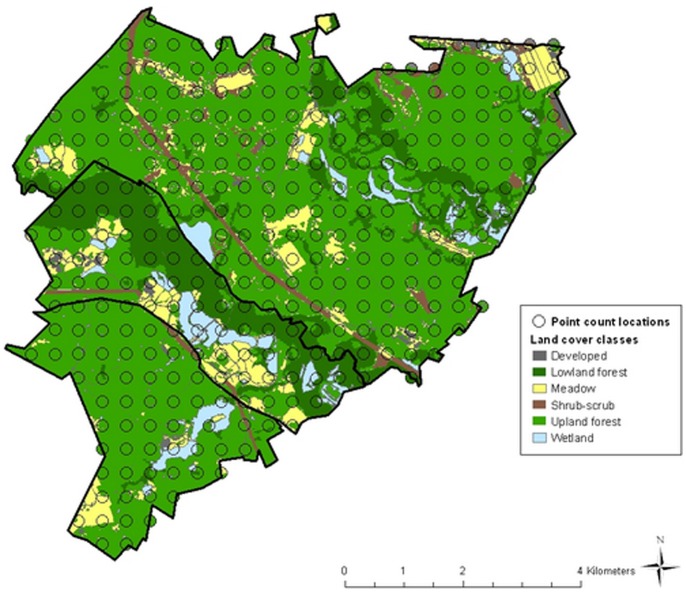
Map of 100-m radius buffers surrounding a grid of point-count stations throughout the Patuxent Research Refuge in Laurel, Maryland. Landcover is modified from the 1992 National Landcover Data set [24].

The Patuxent Research Refuge is located in Prince Georges County, Maryland in the Mid Atlantic Piedmont USA. The 52 km^2^ area of the refuge is primarily deciduous forest, with interspersed meadows, wetlands, and shrub-scrub areas that are managed to provide wildlife habitat. The refuge is gridded at 400-m intervals with permanent markers, and we selected a systematic sample of 316 grid points as locations for point counts (Fig. 1). Points that could not be accessed or were adjacent to a major highway on the western park boundary were not surveyed.

### 2. Field Data Collection

Counts were conducted between 31 May to 4 July 2008 by experienced surveyors. A consistent protocol was followed in counting and recording, in which the observer stood at the point and recorded all birds seen or heard within a 100-m radius for a 5-min count interval. Sites were surveyed 1–7 times (mean = 2.54). Additional details on the avian sampling methods have been published elsewhere [23].

### 3. Landscape Metrics

The 1992 National Land Cover Data set (NLCD) [24] was used to describe the land cover classes on the refuge at a 30-m resolution. The NLCD habitat information was updated based on aerial photographs taken in 2007 [25] and polygons of meadows and wetlands obtained from refuge staff. We defined the following six landcover classes that likely influence bird species’ distributions on the refuge: deciduous, evergreen, and mixed forest (“upland forest”); deciduous and mixed forested wetlands (“lowland forest”); shrub-scrub; meadows; freshwater impoundments and herbaceous wetlands (“wetlands”); and developed areas (Fig. 1). We calculated the proportion of area of each land cover class within 100 m of each point-count location (Table 1).

**Table 1:** Landcover proportions within 100 m of 316 point-count locations throughout the Patuxent Research Refuge, Laurel, Maryland, USA.

Landcover class	Description^a^	Proportion^b^	
Shrub-scrub	Early-successional, transitional, and shrubland habitat	0.030±0.109	(0−1.00)
Meadow	Meadows, pasture/hay, urban/recreational grasses, row crops, and developed open space	0.073±0.192	(0−0.98)
Wetland	Emergent herbaceous wetlands and ponds	0.034±0.123	(0−0.87)
Upland forest	Deciduous, evergreen, or mixed upland forest	0.675±0.366	(0−1.00)
Lowland forest	Seasonally flooded, deciduous or mixed woody wetlands	0.160±0.306	(0−1.00)
Developed	Medium intensity developed areas and roads	0.028±0.066	(0−0.67)

a Modified from the 1992 National Landcover Data set classifications [24].

b Mean +/− standard deviation (range).

### 4. Model Construction

We used a state-space approach to model species distributions, which included an occupancy process model and an observation model of detections [3], [20], [26]. The process model consists of the latent binary occupancy state 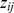 (where 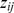 = 1 if species *i* is present at site *j* and zero otherwise) for each of *N* species at *S* sites:
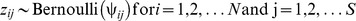



where 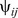 is the probability of occupancy for species *i* in site *j*. The observation model, which is conditional on the corresponding occupancy state, is described as follows:
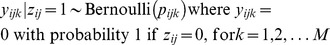
and 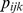 is the probability of detection for species *i* in site *j* (conditional on presence) during *k* of *M >1* visits across which we assume closure [22]. To account for spatial autocorrelation, we included an autologistic variable [27] that is specified as follows:
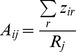



where 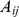 is the proportion of sites, neighboring *j*, that are occupied by species *i* estimated by dividing the total number of presences (in each of the r neighboring cells) of species i, denoted 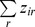, by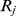, the total number of sites that neighbor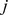. Thus all neighboring sites are given equal weight. We defined the neighborhood using survey sites within 600 m of one another. Using this radius there were 164 sites with 8 neighbors, and all sites, except for two, had three or more neighbors. Eight sites had 9 neighbors due to one irregular sampling location within the grid.

For our global occupancy model, we incorporated six species-specific random effects (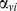; where 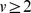 indexes regression parameters); including an intercept term for species (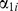) in addition to the autologistic variable followed by five land-cover variables that accounted for variation among species regarding their effects on occupancy:
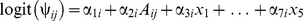



Specifically, we included the following landcover variables: percentage of habitat within a 100 meter radius of lowland forest, developed, wetland, meadow, and shrub-scrub. We excluded upland forest from the set of predictors to avoid multicollinearity, and as such upland forest influence was captured in the intercept term. We confirmed that the remaining land-cover variables had pairwise correlations with absolute values <0.25. We specified vague Bayesian priors for the species-specific logit-scale random effects in the global occupancy model as follows:
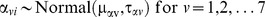





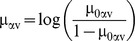


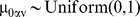





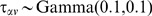



where *v* indexes regression parameters for the global occupancy model, 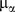 and 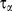 are the logit-scale priors for the mean and precision (i.e., inverse of the variance) of the normal distribution for the random effect, 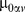 is the prior mean on the probability scale based on a uniform distribution. We assumed a gamma distribution as a prior for 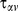 with shape parameters both equal to 0.1.

For our observation model, we included a random effect for species:
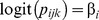



As with the global model, we specified vague Bayesian priors for the species-specific logit-scale random effect in the observation model using a similar approach:
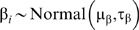





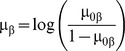


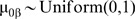





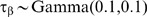



This random-effects modeling approach allows for a large number of species to be analyzed simultaneously rather than in separate models. Using this multi-species modeling approach, precision of occupancy estimates and covariates is improved especially for species that have low detection probability [3]. Predicted occupancy parameters for rarely observed species gravitate toward the means of parameters across the entire assemblage based on an assumption that these difficult-to-detect species follow similar patterns as other species in the community.

In addition to this global model, we constructed three subset models of occupancy to evaluate alternative hypotheses about the relative contributions of spatial autocorrelation and landscape heterogeneity for species distributions. The subset models differed based on inclusion or exclusion of the landcover and autologistic variables for predicting occupancy. The full candidate set of models was as follows: null (neither landcover nor autologistic), landcover (i.e., exogenous landcover only), autologisic (endogenous spatial autocorrelation only), and global (exogenous landcover and endogenous spatial autocorrelation).

### 5. Model Fitting and Analysis

We used WinBUGS version 1.4.3 [28] to fit the four alternative state-space models to the bird detection data, which uses Markov chain Monte Carlo (MCMC) methods. We obtained three independent chains of 20,000 MCMC iterations for each model, from which we discarded the initial 10,000 iterations as “burn-in” and then thinned the remainder by 10 to render 1000 iterations for each parameter’s posterior distribution (e.g., the posterior distribution consisted of a total of 3000 estimates for each parameter). We confirmed convergence using the Gelman-Rubin diagnostic where all parameters had an autocorrelation indicator below 1.1 [29]. This large number of iterations was required due to a high level of autocorrelation among MCMC iterations under the global model, which included effects for spatial autocorrelation and landcover variables in addition to species-level random effects for occupancy and detectability. We also calculated Moran’s I for each of the covariates in the landcover model to determine if they were spatially autocorrelated. We used the same neighborhood structure as that used in the auto-logistic model to maintain the same spatial scale.

### 6. Model Evaluation

We evaluated model performance by computing the area under the curve of the receiver operating characteristic (AUC) [30], [31]. When applied to the dataset used during model construction, AUC measures a model’s goodness-of-fit by estimating the probability that a randomly chosen occupied sampling point (where 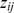 = 1) has a higher probability of occupancy than a randomly chosen unoccupied sampling point (where 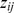 = 0). If a model fits well, then it consistently predicts a higher probability of occupancy for occupied sites yielding an AUC closer to 1.0. Conversely, if a model fits poorly, it will perform the same as chance yielding an AUC closer to 0.5.

We utilized AUC for evaluating our models in two ways. First, we calculated mean and 95% Bayesian credibility interval (BCI) AUC values reflecting goodness-of-fit for each of the four models based on the vector of AUC values across MCMC iterations (henceforth, consolidated AUC values) for all species combined. Second, we calculated AUC values reflecting model goodness-of-fit for each species under each model rendering a mean and 95% BCI AUC value for each species-model combination (henceforth, species-specific AUC values) [32]. Calculating AUCs for each species in each model is made possible by examining the species-specific binary occupancy predictions along with predicted occupancy probabilities for each sampling point across the respective vectors of MCMC iterations. We concluded a statistically significant difference between posterior distributions when the 95% BCI (2.5^th^ to 97.5^th^ percentile of the posterior distribution) for one posterior excluded the BCI of the opposing posterior. When making inferences about the strength of spatial autocorrelation at the individual species level, we examined the log-odds ratio from the autologistic parameter for each species calculated as: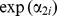. For this analysis, we interpret an odds ratio as the proportional increase in local occupancy probability for each additional neighboring sampling location that is occupied by a conspecific.

## Results

Our analysis considered 90 species of breeding birds that were observed on the refuge (Table 2). Details of the occupancy, detectability, and habitat associations of the species are presented elsewhere [23]. In general, habitat associations of species followed predictable patterns based on the life history and known habitat preferences of the species; i.e., scrub and grassland birds had negative associations with forest habitats, and woodland birds had positive associations with forest habitats (Table 2). All of the landcover covariates exhibited spatial autocorrelation at a local scale (p<0.001 using a Moran’s I calculation for each covariate). Our results focus on relative importance of local landcover and spatial autocorrelation for predicting bird species distributions.

**Table 2:** Landcover associations and spatial autocorrelation under alternative models for distributions of bird species observed during point counts on the Patuxent Wildlife Research Refuge, Maryland.

Common name	Code	Scientific name	Num. detections	Developed	Lowland forest	Meadow	Wetland	Scrub-shrub	Spatial
Canada Goose	CANG	*Branta canadensis*	18	0,0	0,0	0,0	0,0	0,0	18
Wood Duck	WODU	*Aix sponsa*	6	/	/	/	/	/	6
American Black Duck	ABDU	*Anas rubripes*	1	/	/	/	/	/	1
Mallard	MALL	*Anas platyrhynchos*	1	/	/	/	/	/	1
Northern Bobwhite^a^	NOBO	*Colinus virginianus*	3	/	/	/	/	/	3
Wild Turkey	WITU	*Meleagris gallopavo*	5	/	/	/	/	/	5
Double-crested Cormorant	DCCO	*Phalacrocorax auritus*	1	/	/	/	/	/	1
Great Blue Heron	GBHE	*Ardea herodias*	10	0,0	+,+	0,0	0,0	0,0	10
Great Egret	GREG	*Ardea alba*	1	/	/	/	/	/	1
Green Heron	GRHE	*Butorides virescens*	5	/	/	/	/	/	5
Black Vulture	BLVU	*Coragyps atratus*	1	/	/	/	/	/	1
Turkey Vulture	TUVU	*Cathartes aura*	1	/	/	/	/	/	1
Cooper’s Hawk	COHA	*Accipiter cooperii*	3	/	/	/	/	/	3
Red-shouldered Hawk	RSHA	*Buteo lineatus*	12	0,0	0,0	0,0	0,0	0,0	12
Red-tailed Hawk	RTHA	*Buteo jamaicensis*	7	/	/	/	/	/	7
King Rail	KIRA	*Rallus elegans*	1	/	/	/	/	/	1
Killdeer	KILL	*Charadrius vociferus*	8	/	/	/	/	/	8
Rock Pigeon	ROPI	*Columba livia*	1	/	/	/	/	/	1
Mourning Dove	MODO	*Zenaida macroura*	134	0,0	−,0	0,0	0,0	0,0	134
Yellow-billed Cuckoo	YBCU	*Coccyzus americanus*	79	0,0	0,0	0,0	0,0	0,0	79
Barred Owl	BADO	*Strix varia*	1	/	/	/	/	/	1
Chimney Swift^a^	CHSW	*Chaetura pelagica*	9	/	/	/	/	/	9
Ruby-throated Hummingbird	RTHU	*Archilochus colubris*	10	0,0	0,0	0,0	0,0	0,0	10
Belted Kingfisher	BEKI	*Megaceryle alcyon*	1	/	/	/	/	/	1
Red-bellied Woodpecker	RBWO	*Melanerpes carolinus*	181	0,0	+,+	0,0	0,0	−,−	181
Downy Woodpecker	DOWO	*Picoides pubescens*	122	0,0	0,0	0,0	0,0	0,0	122
Hairy Woodpecker	HAWO	*Picoides villosus*	39	0,0	0,0	0,0	0,0	0,0	39
Yellow-shafted Flicker	YSFL	*Colaptes a. auratus*	29	0,0	0,0	0,0	0,0	0,0	29
Pileated Woodpecker	PIWO	*Dryocopus pileatus*	85	0,0	0,0	0,0	0,0	0,0	85
Eastern Wood-Pewee	EAWP	*Contopus virens*	223	0,0	+,0	0,0	0,0	0,0	223
Acadian Flycatcher	ACFL	*Empidonax virescens*	357	−,−	+,+	−,−	−,−	0,0	357
Eastern Phoebe	EAPH	*Sayornis phoebe*	16	0,0	0,0	+,+	+,+	0,0	16
Great Crested Flycatcher	GCFL	*Myiarchus crinitus*	76	0,0	0,0	0,0	0,0	0,0	76
Eastern Kingbird	EAKI	*Tyrannus tyrannus*	21	+,0	0,0	+,+	+,+	+,+	21
White-eyed Vireo	WEVI	*Vireo griseus*	74	0,0	0,0	+,+	+,+	+,+	74
Yellow-throated Vireo	YTVI	*Vireo flavifrons*	97	0,0	+,+	0,0	0,0	0,0	97
Warbling Vireo	WAVI	*Vireo gilvus*	1	/	/	/	/	/	1
Red-eyed Vireo	REVI	*Vireo olivaceus*	549	0,0	+,+	−,−	−,−	0,0	549
Blue Jay	BLJA	*Cyanocitta cristata*	205	0,0	−,0	0,0	0,0	+,0	205
American Crow	AMCR	*Corvus brachyrhynchos*	113	0,0	+,0	0,0	0,0	+,0	113
Fish Crow	FICR	*Corvus ossifragus*	4	/	/	/	/	/	4
Purple Martin	PUMA	*Progne subis*	1	/	/	/	/	/	1
Tree Swallow	TRES	*Tachycineta bicolor*	30	0,0	0,0	+,+	+,+	0,0	30
Northern Rough-winged Swallow	NRWS	*Stelgidopteryx serripennis*	2	/	/	/	/	/	2
Barn Swallow	BARS	*Hirundo rustica*	8	/	/	/	/	/	8
Carolina Chickadee	CACH	*Poecile carolinensis*	296	0,0	0,0	0,0	0,0	0,0	296
Tufted Titmouse	TUTI	*Baeolophus bicolor*	491	0,0	0,0	0,0	0,0	0,0	491
White-breasted Nuthatch	WBNU	*Sitta carolinensis*	189	0,0	+,+	0,0	0,0	0,0	189
Carolina Wren	CARW	*Thryothorus ludovicianus*	223	+,0	+,+	+,+	+,+	0,0	223
House Wren	HOWR	*Troglodytes aedon*	15	0,0	0,0	+,+	+,+	+,0	15
Blue-gray Gnatcatcher	BGGN	*Polioptila caerulea*	177	0,0	+,0	0,0	0,0	0,0	177
Eastern Bluebird	EABL	*Sialia sialis*	62	0,0	0,0	+,+	+,+	0,0	62
Wood Thrush^b^	WOTH	*Hylocichla mustelina*	347	−,−	0,0	−,0	−,0	0,0	347
American Robin	AMRO	*Turdus migratorius*	152	0,0	0,0	+,+	+,+	+,+	152
Gray Catbird	GRCA	*Dumetella carolinensis*	36	+,+	0,0	+,0	+,0	+,+	36
Northern Mockingbird	NOMO	*Mimus polyglottos*	52	0,0	0,0	+,+	+,+	+,+	52
Brown Thrasher	BRTH	*Toxostoma rufum*	16	0,0	0,0	0,0	0,0	+,+	16
European Starling	EUST	*Sturnus vulgaris*	20	+,0	0,0	+,+	+,+	+,+	20
Cedar Waxwing	CEDW	*Bombycilla cedrorum*	23	0,0	0,0	+,+	+,+	0,0	23
Northern Parula	NOPA	*Parula americana*	88	0,0	+,+	+,+	+,+	0,0	88
Pine Warbler	PIWA	*Dendroica pinus*	141	0,0	−,−	−,0	−,0	−,−	141
Prairie Warbler^b^	PRAW	*Dendroica discolor*	48	0,0	0,0	+,+	+,+	+,+	48
Black-and-white Warbler	BAWW	*Mniotilta varia*	9	/	/	/	/	/	9
American Redstart	AMRE	*Setophaga ruticilla*	54	0,0	+,+	0,0	0,0	0,0	54
Prothonotary Warbler^b^	PROW	*Protonotaria citrea*	16	0,0	+,+	0,0	0,0	0,0	16
Worm-eating Warbler^b^	WEWA	*Helmitheros vermivorum*	13	0,0	0,0	0,0	0,0	0,0	13
Ovenbird	OVEN	*Seiurus aurocapilla*	407	−,0	−,−	−,−	−,−	−,−	407
Louisiana Waterthrush	LOWA	*Parkesia motacilla*	18	0,0	+,+	0,0	0,0	0,0	18
Kentucky Warbler	KEWA	*Oporornis formosus*	29	0,0	0,0	0,0	0,0	0,0	29
Common Yellowthroat	COYE	*Geothlypis trichas*	88	0,0	+,+	+,+	+,+	+,+	88
Hooded Warbler	HOWA	*Wilsonia citrina*	102	−,0	0,0	−,0	−,0	0,0	102
Yellow-breasted Chat	YBCH	*Icteria virens*	57	0,0	0,0	+,+	+,+	+,+	57
Eastern Towhee^a^	EATO	*Pipilo erythrophthalmus*	129	+,+	−,−	+,+	+,+	+,+	129
Chipping Sparrow	CHSP	*Spizella passerina*	49	+,0	0,0	+,+	+,+	+,0	49
Field Sparrow	FISP	*Spizella pusilla*	76	0,0	0,0	+,+	+,+	+,+	76
Grasshopper Sparrow	GRSP	*Ammodramus savannarum*	4	/	/	/	/	/	4
Song Sparrow	SOSP	*Melospiza melodia*	9	/	/	/	/	/	9
Summer Tanager	SUTA	*Piranga rubra*	35	0,0	0,0	0,0	0,0	+,+	35
Scarlet Tanager	SCTA	*Piranga olivacea*	232	0,0	0,0	−,0	−,0	0,0	232
Northern Cardinal	NOCA	*Cardinalis cardinalis*	236	+,0	+,0	+,+	+,+	+,+	236
Blue Grosbeak	BLGR	*Passerina caerulea*	47	0,0	0,0	+,+	+,+	+,+	47
Indigo Bunting	INBU	*Passerina cyanea*	137	+,+	0,0	+,+	+,+	+,+	137
Red-winged Blackbird	RWBL	*Agelaius phoeniceus*	59	+,+	0,0	+,+	+,+	0,0	59
Eastern Meadowlark	EAME	*Sturnella magna*	6	/	/	/	/	/	6
Common Grackle	COGR	*Quiscalus quiscula*	53	+,0	0,0	+,+	+,+	0,0	53
Brown-headed Cowbird	BHCO	*Molothrus ater*	96	0,0	0,0	0,0	0,0	0,0	96
Orchard Oriole	OROR	*Icterus spurius*	37	+,0	0,0	+,+	+,+	0,0	37
Baltimore Oriole	BAOR	*Icterus galbula*	6	/	/	/	/	/	6
House Finch	HOFI	*Carpodacus mexicanus*	2	/	/	/	/	/	2
American Goldfinch	AMGO	*Spinus tristis*	164	0,0	0,0	+,+	+,+	0,0	164

a Species of high regional conservation concern [18].

b Species of high continental conservation concern [18].

Associations for species with fewer than 10 detections are not shown and instead indicated by slashes (/); effects for these species are potentially misleading due to the lack of information. Effects reported for the landcover variables are from one model with only land cover variables, followed by another model that also includes spatial autocorrelation (the global model). Effects reported for the spatial autocorrelation (autologistic) variable are from one model with only spatial autocorrelation, followed by another model that also includes land cover variables (the global model). Directions of effects based 95% BCIs:+ = above zero, − = below zero, 0 = includes zero. See Table 1 for descriptions of landcover variables.

### 1. Model Evaluation

**Figure 2:**
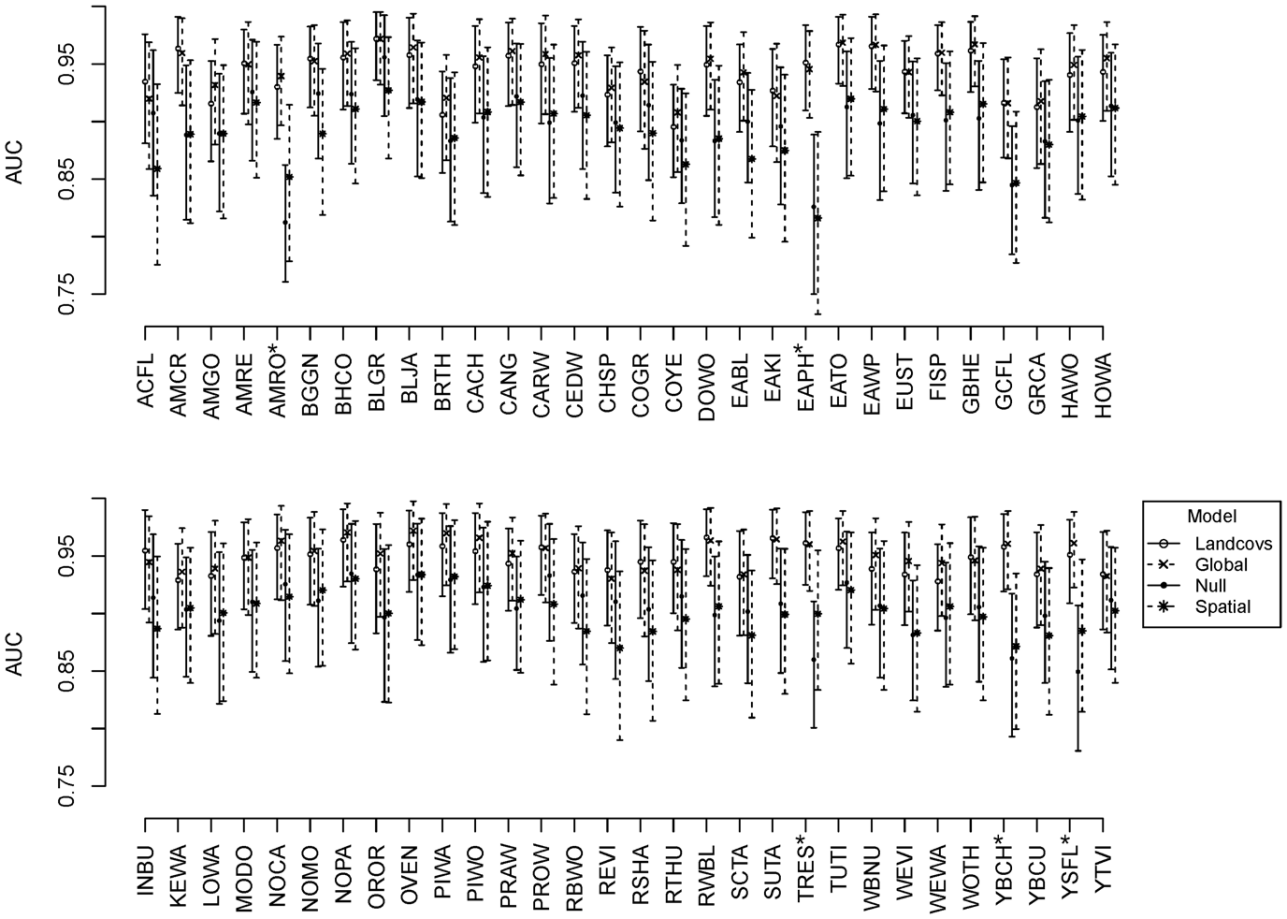
Fit of alternative models describing distributions of bird species sampled on the Patuxent Wildlife Research Refuge. Models included the following: null = species-specific occupancy, assumed to be constant across all sampling locations, landcovs = six landcover covariates for species-specific occupancy, spatial = autologistic covariate for species-specific occupancy, and global = landcovs and spatial combined. All models account for imperfect detection of species. Symbols represent the mean AUC values and whiskers represent the 95% Bayesian credibility intervals (BCIs) of the AUC estimates. An asterisk (*) following the species code indicates that the landcover model had significantly better fit than the null model.

Based on the consolidated community-level AUC values, the landcover model (mean: 0.942; BCI: 0.934–0.950) and global model (0.945; 0.935–0.954) performed similarly and had significantly better fits than the null (0.882; 0.865–0.899) and autologistic models (0.887; 0.873–0.901), which were themselves quite similar. We refer to the landcover model as the top model because of its parsimony (i.e., fewer parameters but nearly identical fit to the global model). Species-specific AUC values revealed comparable patterns, with the landcover model having similar AUC values to the global model and the spatial model having similar AUC values to the null model (Fig. 2 Every species had higher mean AUCs in the models that contained the landcover covariates as compared to the null and autologistic models (Fig. 2), though most of these differences were not statistically significant. For 5 of the 90 species the landcover model had significantly better fit than the null model. Model fit was very good across species and models, as the means of the AUC posteriors ranged from 0.776 to 0.977 and the BCIs spanned 0.712 −0.997.

### 2. Landcover Effects

With the exception of percent developed, there were few cases where the landcover model revealed a significant association and the global model did not for a given landcover type (Table 3). There was perfect agreement between the landcover and global models regarding species associations with meadow and wetland when the effect was positive, with a single exception of gray catbird. For cases where the models agreed about positive associations, the most frequent significant associations across the community were for the natural open landcover types (28% of species, n = 25 each) followed by scrub-shrub (18%, n = 16), lowland forest (13%, n = 12), and developed (4%, n = 4). A few species showed negative associations under both models (2–3%, n = 2 to 3): Acadian flycatcher, downy woodpecker, eastern towhee, ovenbird, pine warbler, red-bellied woodpecker, red-eyed vireo, and wood thrush (Table 2). Most species that showed significant association with percent developed in the landcover model did not show this association in the global model. Similarly, the global model showed no significant association for most species that had exhibited a negative association with lowland forest, meadow, or wetland in the landcover model. Throughout the avian community, there were no cases where the global model showed a significant association but the landcover model did not. Likewise when there was a significant association under both the landcover and global models, they always agreed on the direction of the effect.

**Table 3:** Numbers of species with significant associations between occupancy and landcover variables under two alternative models (landcover only and global models) based on point count surveys throughout the Patuxent Wildlife Research Refuge, Maryland, USA.

Criterion^a^	Developed	Lowland forest	Meadow	Wetland	Scrub-shrub
Both models positive	4	12	25	25	16
Both models negative	2	3	3	3	3
Only reduced model positive	7	4	1	1	4
Only reduced model negative	2	2	4	4	0

a Global model included both landcover and spatial covariates; reduced model included only landcover covariates.

### 3. Spatial Effects

**Figure 3:**
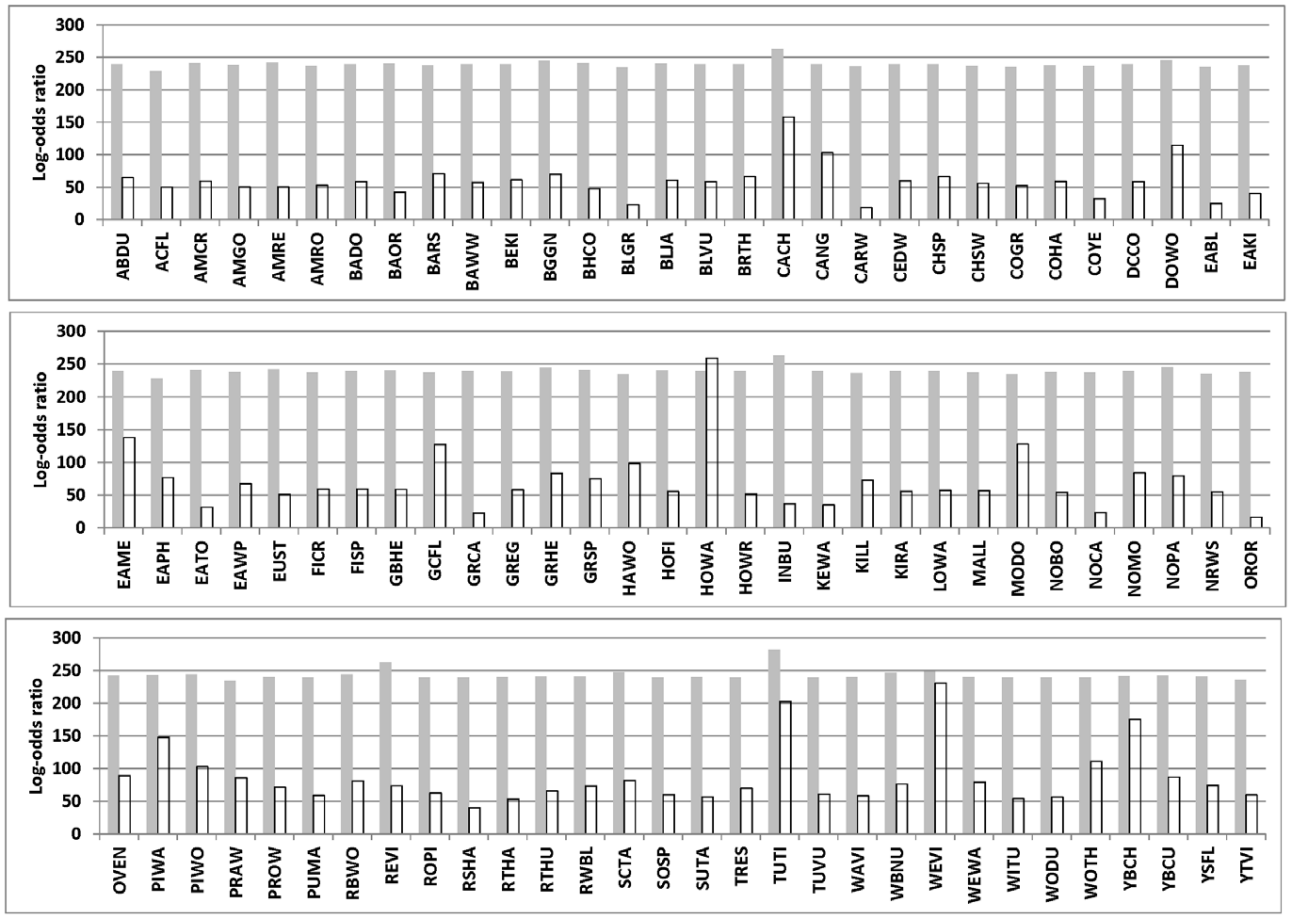
Strength of autocorrelation for individual species across the bird community at Patuxent National Wildlife Refuge. Gray bars represent a reduced model with only spatial effects included; unfilled bars represent a global model with landcover and spatial effects included. Bar heights represent means from the posterior distribution of a Bayesian analysis based on three independent MCMC chains (n = 20,000). All of the Bayesian credibility intervals overlapped and are not shown for clarity.

All species exhibited statistically significant positive spatial autocorrelation in both the global and spatial-only models (i.e., 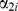
>0). Effect sizes of the autologistic parameter were high and statistically indistinguishable among species in the spatial-only model (range of means as log-odds ratios: 228 to 281; range of their BCI widths: 170 to 219; Fig. 3). When compared to the spatial-only model, means of the autologistic parameter from the global model yielded greater variation among species with respect to the autologistic parameter (range of means as log-odds ratios: 16.4 to 259; range of their BCI widths: 88.3 to 2236). None of the species-specific differences between models with respect to the relative magnitude of the autologistic parameter were significant in that the BCIs overlapped. Based on the BCIs of this autologistic parameter that excluded means of opposing species in the global model, hooded warbler and white-eyed vireo tended to have the strongest spatial autocorrelation whereas species tending to have the weakest spatial autocorrelation included orchard oriole, Carolina wren, gray catbird, blue grosbeak, northern cardinal, eastern bluebird, eastern towhee and common yellowthroat.

## Discussion

We found that landcover heterogeneity was more important for understanding local-scale species distributions than was spatial autocorrelation alone or a null model that assumed a homogeneous distribution of species for an entire avian community. When focusing on the distribution of individual species, accounting for spatial autocorrelation alone had lower AUC values (and thus a poor fit to the data) than when accounting for landcover heterogeneity alone. This may in part be due to the fact that all of the landcover covariates were spatially correlated and thus might also explain spatial correlation in the species distributions. This notion is supported by our unpublished findings that the residuals for most species in the landcover model were not spatially autocorrelated. The landcover hypothesis therefore, was more consistent with our results than was the aggregation hypothesis for explaining local-scale individual species distributions.

In our study there were numerous significant associations between specific landcover variables and patterns of individual species occupancy that were in agreement between models with and without the spatial component. We found that in both the landcover and global models, species distributions were often driven by associations with undeveloped openings (i.e., meadows and wetlands). The global and landcover-only models disagreed more frequently when predicting relationships between species distribution and the amount of developed land. There was considerable uncertainty whether landscape heterogeneity alone or combined with spatial autocorrelation better reflected species distributions. Although we found consistent evidence for particular species associations with vegetated landcover (i.e., meadow, wetland, scrub-shrub, and lowland forest) between landcover models with and without spatial autocorrelation, the apparent strong and consistent evidence for conspecific aggregation across the bird community weakened for all but very few species after accounting for landcover effects (Fig. 3). Throughout the analysis, we accounted for potential confounding factors of heterogeneity among individual species occupancy and detection, which enabled robust inferences about the relative value of alternative sources of spatial heterogeneity for predicting the distribution of species across a community. To our knowledge, this represents the first attempt to reconcile the relative and combined influence of endogenous and exogenous drivers at a local scale for individual species across an entire bird community.

An emphasis of our study was to better understand interactions between individual bird species distributions and various types of openings within a landscape dominated by contiguous forest. Across the bird community on the refuge, meadows and wetlands were important drivers for a number of species regardless of whether we accounted for spatial autocorrelation. Although the landcover model revealed a significant negative relationship between developed landcover and occupancy of several species, this effect dissipated when accounting for spatial autocorrelation. A possible explanation is that the sparse but highly aggregated distribution of developed lands on the refuge induces spatial autocorrelation in the associated bird species rather than endogenous factors alone. In contrast, species associations with vegetated cover types were relatively consistent whether or not spatial autocorrelation was included in the model. We hypothesize that at a local scale, intrinsic and extrinsic factors interact to generate distributions of species associated with vegetated cover types whereas aggregation of synanthropic species is largely driven by exogenous factors. Such hypotheses about interactions between spatial arrangements of habitat and mobile species, though conceptually familiar [12], [33], have rarely been examined rigorously [4].

To our knowledge, few studies investigating distributions of multiple forest bird species have accounted for spatial autocorrelation [4], [6], [34], [35]. Investigating 50–400 km neighborhoods surrounding focal observations of over 100 bird species across North America, Bahn et al. [6] found strong evidence for spatial autocorrelation with respect to the proportion of years a conspecific was observed within the neighborhood during a 10-year period based on conditional autoregression. When analyzing counts for three breeding passerine species in the Appalachian Mountains, Lichstein et al. [4] found evidence of decreasing spatial autocorrelation from distances ranging from 150 m to 1500 m at 150-m intervals even after incorporating broad-scale spatial trend and environmental covariates. They found that much of the spatial autocorrelation in bird counts was attributed to spatial autocorrelation in landcover variables. Likewise, Betts et al. [34] found evidence of spatial autocorrelation between 350 and 700 m and then diminishing out to 3500 m across the 23 species examined in New Brunswick. Clearly, birds exhibit evidence of conspecific aggregation at multiple spatial scales. Finally, Thogmartin and Knutson [36] reported spatial dependence at 800 m for counts of three forest species in north-central US despite inclusion of landscape metrics at multiple scales ranging from 0.1 to 10-km.

Across these studies and our own, we hypothesize that bird species breeding in forested landscapes exhibit spatial aggregation within fine (100–800 m) and coarse (>10^4^ m) neighborhoods but less so within an intermediate (10^3^–10^4^ m) neighborhood. This hypothesis is supported by some of our own unpublished analyses, which indicate that the strength and consistency of spatial autocorrelation across the avian community declines when considering a 10^3^ neighborhood. We suggest that mechanisms giving rise to aggregation of conspecific mobile species at fine scales likely contrast with those at coarse scales. At a fine scale, aggregation of landcover characteristics to which species are adapted in addition to opportunities for exchange of genetic material and information about local food resources are likely powerful drivers of conspecific clustering, and certain life history strategies emerge from the particular mechanisms for this fine-scaled clustering. At coarser scales, geomorphic landforms and climatic envelopes likely drive neighborhood associations that dictate the geographic ranges of species. As highly mobile organisms, bird species may fail to exhibit spatial structuring at intermediate scales within which dispersal is less frequent, information flows are localized, and landcover types become spatially diversified and therefore exhibit little aggregation. This diminished clustering at intermediate scales has been observed in plant species [8], but to our knowledge a comprehensive cross-scale analysis has yet to be conducted for mobile species. A multi-scale investigation would be necessary to evaluate this intermediate-scale spatial-structure hypothesis.

Spatial resolution is a critical consideration when examining patterns of species distributions [8], [9]. Unless the resolution (i.e., distance between sample units) exceeds the home range size of a mobile species, aggregations of occurrences may simply represent individual movements rather than actual aggregation of individuals among adjacent patches (i.e., sample units). For example, a wide-ranging species may occur across multiple sample units, whereas individual territories held by this species may be diffusely distributed themselves. Therefore, the resolution is an integral component when making inferences about patterns of species occurrences. The 400-m (16-ha) grid sampling design in our study encompasses typical home range sizes for non-raptorial breeding bird species [37], [38], with the exception of larger-bodied woodpeckers and crows [39]–[42]. Evidence of spatial aggregation of these wide-ranging species at a local scale could therefore be explained by within season dispersal of individuals.

In addition to spatial resolution, we must also take into account other scopes of inference when interpreting results from our study. First, we investigated the bird community within the Patuxent Research Refuge, which should be representative of the more natural areas of the Mid Atlantic Piedmont but some care must be taken when applying these findings beyond the Refuge. Much of the original forest and wetlands in this ecoregion has been converted to agricultural and residential land uses [18], and bird community composition and distribution will of course differ in more fragmented landscapes [43] with conspecific aggregation perhaps becoming more pronounced. Conducting a validation of this model with a similar dataset outside the refuge would reveal its ability to predict outside of this system. Second, our study took place during a single breeding season and therefore provides only a snapshot of the bird community. A multi-season sample would offer the opportunity to investigate temporal dynamics in species occupancy and perhaps a more comprehensive representation of bird-habitat associations and aggregations [3], [21]. Third, we tried fitting a conditional autoregressive (CAR) model [44] to our dataset, but unfortunately the model failed to converge for many of the species despite several attempts to adjust and restrict the prior distributions. The CAR model incorporates spatial dependence as a random effect for each sampling location as opposed to the autologistic which assumes that the spatial effect is fixed. It is possible that a greater number of detections for some species and/or sampling points than was available in our study could enable convergence of the CAR model. The CAR model is more flexible than the autologistic, and practitioners should consider this approach as it will allow for examination of residual spatial autocorrelation providing additional information about the relative influence of endogenous and exogenous factors [6].

Fourth, we examined associations between bird species occupancy and local landcover percentages but not measures of landscape configuration like edges, core areas, or patch size, which have long been recognized as important drivers of bird species distributions [45], [46]. Furthermore, we investigated only linear effects of landcover, whereas the effects may indeed be curvilinear in some cases. We therefore cannot make any conclusions about the linearity of landcover associations nor the relative influence of these broader-scale landscape patterns. A larger set of grid points, however, would offer sufficient sample space to explore a broader suite of landscape metrics in addition to conspecific aggregation and their interactions, therefore providing greater insights into associations between land cover characteristics and bird species distributions.

Finally we used AUC to evaluate and compare model fit rather than one of the information-theoretic model-selection criteria [47], [48], which would allow us to make inference about the relative parsimony and weights of evidence among our alternative occupancy models. Standard model selection criteria (such as AIC) unfortunately have inherent biases that confound inferences about hierarchical models, such as our multi-species occupancy model [49], [50], whereas AUC can provide a more straightforward estimate of model fit [30]. Applying robust and computing-intensive model-selection approaches, such as reversible-jump MCMC [49], to compare spatial community models is an area of future research.

Birds are not only a useful taxon for studying species distributions, but they also present opportunities to account for imperfect detection within a community-level analysis. With regard to imperfect detection, there are at least two reasons why a bird species may be present but not detected [51]. First, as mobile species, they may be available for detection at a given location during some times but not during others. Second, they may be available for detection but go unseen and unheard by an observer. Imperfect detection is therefore a potentially important source of bias to consider when investigating drivers of bird species distributions. On the other hand, breeding bird species assemblages offer an opportunity to conduct community-wide analyses that enable us to examine species-specific occupancy and detectability by sharing detection information across species through hierarchical, random-effects modeling [3].

In this paper we have addressed hypotheses about interactions between bird species distributions and landcover characteristics at a local scale while accounting for potentially confounding factors of partial observability, spatial autocorrelation, and shared traits among species in the community. We have also proposed an intermediate-spacing hypothesis by placing our findings within the context of similar investigations that spanned a broad set of spatial scales. Furthermore, we developed a modeling approach for investigating spatial autocorrelation of conspecific distribution patterns simultaneously for multiple species while accounting for partial observability and allowing for incorporation of habitat covariates. Our approach is an extension of the hierarchical Bayes multi-species occupancy modeling framework by Dorazio et al. [20], which itself is an extension of a single-species modeling approach [22]. Likewise, our hierarchical Bayes spatial community modeling framework can be readily extended to account for interspecific interactions [52], misidentification of species [53], and community dynamics [3]. Such an extensible framework is a powerful tool for examining the complex sets of hypotheses surrounding interactions between species distributions and their environment.
